# Effectiveness of the WHO Protocol for the Management of Shock in Children With Severe Acute Malnutrition

**DOI:** 10.7759/cureus.46252

**Published:** 2023-09-30

**Authors:** Chandan Kumar, Shiva Manwatkar, Anil K Saroj, Tej Bali Singh, Sunil Kumar Rao

**Affiliations:** 1 Division of Pediatric Intensive Care and Pulmonology, Department of Pediatrics, Institute of Medical Sciences, Banaras Hindu University, Varanasi, IND; 2 Department of Preventive Medicine, Institute of Medical Sciences, Banaras Hindu University, Varanasi, IND

**Keywords:** fluid-refractory shock, inotropes, mortality, who, severe acute malnutrition

## Abstract

Background

The WHO protocol for the management of shock in children with severe acute malnutrition (SAM) is not supported by physiological evidence. In this study, we aimed to assess the effectiveness of the WHO treatment protocol in the management of shock in children with SAM.

Methodology

This cohort study included children aged 2-60 months with WHO-defined SAM and fulfilling the WHO criteria for identification of shock. The exclusion criteria included severe anemia (hemoglobin <4 g/dL), congenital anomalies, congenital heart defects, and chronic diseases. The WHO treatment protocol for the management of shock was used, and features of resolution of shock were assessed at eight and 24 hours. Oliguria was recorded at eight and 24 hours along with in-hospital mortality. Multiple logistic regression was used to determine predictors of mortality.

Results

Of 53 children, 40 (75.4%) were discharged and 13 (24.5%) expired. We observed significant resolution of features of shock at 24 hours compared to eight hours (35 (71.4%) vs. 10 (18.8%), p < 0.0001). Further analysis revealed a significant resolution of features of shock (p = 0.03) at 24 hours in both fluid-responsive (24 vs. 10) and fluid-refractory children (11 vs. 27) compared to eight hours. Multivariate analysis revealed that mechanical ventilation was positively related to death (odds ratio (OR) = 85, 95% confidence interval (CI) = 8.49, 860, p < 0.0001), and inotrope scores <20 (OR = 0.053, 95% CI = 0.004, 0.64, p = 0.021) and blood transfusion (OR = 0.025, 95% CI = 0.001, 0.61, p = 0.024) had favorable outcomes.

Conclusions

The WHO protocol for the management of shock in children with SAM is effective in fluid-responsive shock whereas evidence was inconclusive in fluid-refractory shock.

## Introduction

Shock is a major cause of death in children with severe acute malnutrition (SAM), and the current treatment guidelines are based on limited evidence [1-3]. The WHO protocol for the management of shock in children with SAM is not supported by physiological evidence [1] because it recommends restrictive bolus of hypotonic fluid and blood transfusion in cases of fluid-refractory shock. Furthermore, the guidelines are silent when children deteriorate after fluid bolus. Neither restrictive fluid bolus nor blood transfusion replete water and electrolyte loss in severe dehydration but increase the chance of persistent hypovolemia and circulatory failure [2]. There is a paucity of studies evaluating the efficacy of the WHO protocol for the management of shock in children with SAM, and the reported literature shows heterogeneity in methodologies and outcome measures [2-5]. The present study assesses the effectiveness of the WHO protocol for the management of shock in children who fulfilled the WHO criteria for a diagnosis of SAM as well as shock.

## Materials and methods

This cohort study was conducted from September 2018 to May 2020 in a medical college hospital situated in northern India. SAM was defined as a mid-upper arm circumference (MUAC) <11.5 cm, a weight-for-height z-score WHZ (WHZ) <−3, or bilateral pitting edema according to the WHO guidelines. Inclusion criteria were children aged 2-60 months with WHO-defined SAM who fulfilled the WHO criteria of shock, i.e., (i) fast and weak volume pulse (fast pulse rate (PR) defined as >160 in a child aged less than one year and PR >140 in a child aged one to five years), (ii) capillary refill time (CRT) of more than three seconds, (iii) temperature gradient (cooler extremities to warmer central body to touch), and (iv) impaired consciousness [6]. Exclusion criteria included severe anemia (hemoglobin <4 g/dL), congenital anomalies, congenital heart defects, chronic diseases, and organic causes of SAM (tuberculosis, HIV). The study was approved by the Ethics Committee (approval number: Dean/2018/EC/898, dated November 14, 2018) and informed consent was obtained from the parents before the study. Variables recorded were detailed history including sociodemographic profile, clinical examination, anthropometry, laboratory test results, arterial blood gas findings, and culture. Diagnosis, treatment, and complications were recorded in predesigned case report forms. All children were managed as per the WHO-recommended facility-based management of children with SAM [6]. The WHO protocol for the management of shock was used and outcome variables (features of resolution of shock, oliguria, mortality) were recorded at eight and 24 hours.

**Figure 1:**
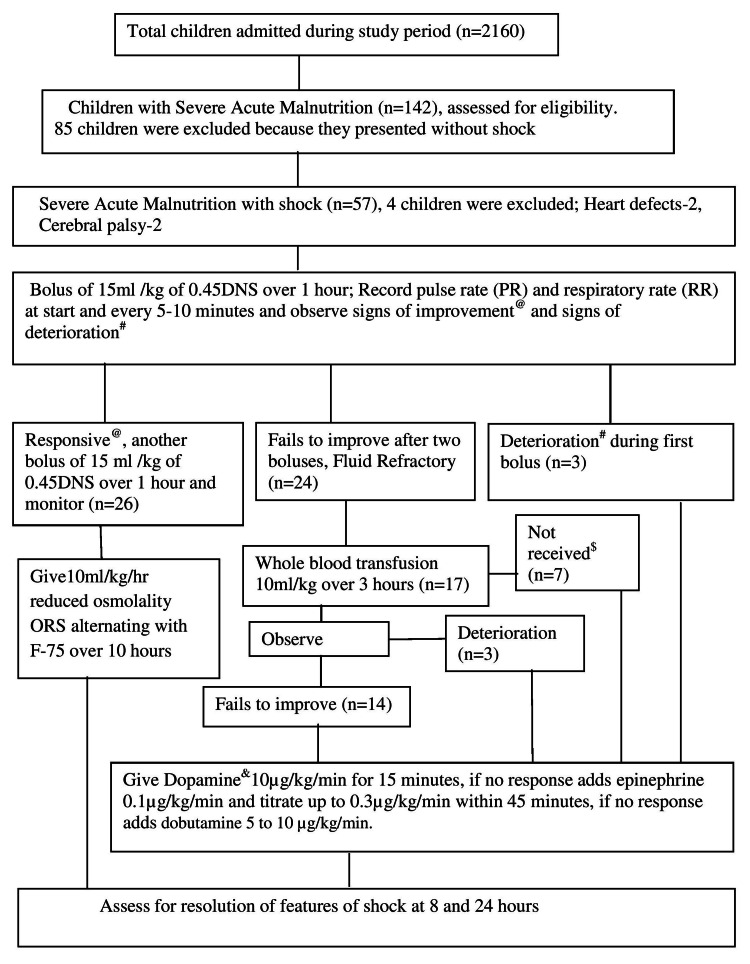
Flow of study showing recruitment of children, intervention received, and response. @: signs of improvement are a fall in pulse rate (PR) and respiratory rate (RR), good pulse volume, and improvement in consciousness; #: signs of deterioration are an increase in PR by 15/minute and RR by 5/minute, tender hepatomegaly, gallop rhythm, engorged neck veins, and bilateral fine crept; $: deviation from protocol; &: the sequence of inotrope used was dopamine, epinephrine, and dobutamine

The WHO protocol for the management of shock is summarized in Figure 1. A fluid bolus of 15 mL/kg body of 0.45% dextrose normal saline (DNS) was given to all recruited children over one hour and PR and respiratory rate (RR) were recorded at the start and then every 5-10 minutes. For children who showed signs of improvement, a second bolus of the same fluid IV 15 mL/kg was given over another hour and categorized as fluid-responsive shock and switched to either oral or nasogastric rehydration with ORS 10 mL/kg/hour alternating with F-75 over 10 hours. If the child failed to improve after two boluses (PR and RR did not fall, low-volume pulse, and no change in consciousness), it was categorized as fluid-refractory shock and the child received a transfusion of whole blood at 10 mL/kg slowly over three hours (maintenance fluid at 4mL/kg/hour 0.45% DNS while awaiting blood). Inotropes were used in children who deteriorated during IV rehydration (increase in RR by 5/minute and PR by 15/minute or more), had engorgement of jugular veins/puffiness of eyes/tender hepatomegaly, did not receive a blood transfusion (deviation from protocol), deteriorated, or did not improve after the blood transfusion. The following definitions were used: the resolution of shock was defined as (i) age-appropriate PR, (ii) CRT <3 seconds, (iii) decreasing trends of lactate and improving trends of pH; (iv) improvement in consciousness; and (v) urine output >1 mL/kg/hour. Mortality was defined as death during the hospital stay. Sepsis was defined as systemic inflammatory response syndrome plus suspicion/presence of infection and/or culture positivity. Inotrope score was calculated as described by Wernovsky et al. [7].

Sample size

Assuming a 25% deviation from the WHO protocol for the management of shock in children with SAM with a study power of 80% with an alpha error of 0.01 and considering 22% mortality at 24 hours (previous data of our pediatric intensive care unit showed 22% mortality at 24 hours in children with SAM and shock), a sample size of 53 children was needed.

Statistical analysis

All data were entered into an Excel sheet and analyzed using SPSS version 21 (IBM Corp., Armonk, NY, USA) The numerical data were represented as mean (SD), median (IQR), frequency (n), and percentage (%). The difference in proportion was compared using the chi-square and Fisher exact test. The Mann-Whitney test was used to compare skewed data presented as median (IQR). A p-value <0.05 was considered significant. Univariate and multiple logistic regression was used to determine predictors of mortality.

## Results

A total of 120 episodes of fluid bolus were recorded during the study period; 52 and 48 episodes in fluid-responsive and fluid-refractory children, respectively, and three episodes in children who showed signs of deterioration and 17 episodes of blood transfusion (Figure 1). Six (5%) episodes of fluid overload (three after the first fluid bolus and three after the blood transfusion) were recorded. Of the 53 children, 32 (60.3%) were male and 21 (39.6%) were female, and the median (IQR) age (months) of the cohort was 13.5 (9-24). Acute diarrhea (40, 75.4%), sepsis (30, 56.6%), and pneumonia (12, 22.6%) were common comorbidities in the cohort. In total, 26 (49%) and 27 (50.9%) children had fluid-responsive and fluid-refractory shock, respectively. The inotrope(s) used in refractory cases were dopamine (all 27 cases), adrenaline (15 cases), and dobutamine (13 cases). The median (IQR) inotrope score was 40 (10-50). The baseline characteristics and interventions are shown in Table 1. Fluid-refractory children were significantly lethargic, oliguric, uremic, septic, and acidotic compared to fluid-responsive children (p < 0.05) (Table 2). We observed that the resolution of features of shock at eight and 24 hours were 10/53 (18.9%) and 35/49 (71.4%), respectively, and the difference in proportion was significant (p < 0.0001) (Table 3). Further analysis revealed there was significant resolution of features of shock (p = 0.03) at 24 hours in both fluid-responsive (24 vs. 10) and fluid-refractory children (11 vs. 27) compared to eight hours. Of the 53 children, 40 (75.4%) were discharged and 13 (24.5%) died. All deaths were observed among fluid-refractory shock cases and we found four (17.3%) cases at 24 hours. All expired children during hospitalization had oliguria, acidosis, and shock. Oliguria was present in 28 (52.8%) children at eight hours and there was a significant decrease in occurrences of oliguria at 24 hours (p = 0.002). On univariate analysis, it was found that edema, oliguria, lethargy, blood transfusion, urea, C-reactive protein, mechanical ventilation, inotrope scores, and pH at admission were significantly associated with mortality (p < 0.05) (Table 4). Multivariate analysis revealed mechanical ventilation was positively related to death (OR = 85, 95% CI = 8.49, 860, p < 0.0001), inotrope scores <20 (OR = 0.053, 95% CI = 0.004, 0.64, p = 0.021) and blood transfusion (OR = 0.025, 95% CI = 0.001, 0.61, p = 0.024) had favorable outcomes. Moreover, the model explained 87% of the contribution to death by these parameters.

**Table 1:** Baseline characteristics of the study cohort. MUAC: mid-upper arm circumference; @: three children developed fluid overload during the first bolus and three children developed fluid overload after the blood transfusion; #: 24 children required a blood transfusion; however, 17 received a blood transfusion; $: the inotrope score was calculated as described by Wernovsky; &: X-ray of the chest was done in 12 children

Variables	Values, total (n = 53)
Age, months, median (IQR)	13.5 (9, 24)
Male, n (%)	32 (60.3)
Anthropometry	
Weight, kg, mean (SD)	6.18 (2.56)
MUAC, cm, mean (SD)	9.84 (1.37)
Clinical parameter	
Edema, n (%)	15 (28.3)
Heart rate, mean (SD)	158.37 (13.54)
Respiratory rate, mean (SD)	55.46 (11.67)
Oliguria, n (%)	28 (52.8)
Fluid-responsive, n (%)	26 (49)
Fluid-refractory, n (%)	24 (50.9)
Fluid overload^@^, n (%)	06 (11.3)
Pediatrics Index of Mortality-3, median (IQR)	8.80 (5.2, 35.4)
Interventions	
Blood Transfusion^#^, n (%)	17 (32)
Inotropes score^$^, median (IQR), n = 27	40 (10, 50)
Mechanical Ventilation (MV), n (%)	10 (18.8)
Duration of MV (hour), median (IQR)	48 (13.75, 78)
Blood biochemistry and investigations	N = 53
Hemoglobin, g/dL, mean (SD)	9.29 (2.03)
Total leukocyte count, cells/mm^3^, median (IQR)	12,700 (8,668.5, 18,500)
C-reactive protein, mg/mL, median (IQR)	10 (3.1,26.6)
Urea, mg/dL, mean (SD)	42.18 (4.01)
Creatinine, mg/dL, median (IQR)	0.50 (0.40, 0.67)
Culture positive sepsis, n (%)	8 (15)
Abnormal Chest X-ray^&^, n (%)	12 (22.6)
Outcomes	
Discharged, n (%)	40 (75.4)
Expired, n (%)	13 (24.5)
Duration of stay in hospital, days, median (IQR)	14 (7, 18)

**Table 2:** Baseline characteristics of fluid-responsive and fluid-refractory children. MUAC: mid-upper arm circumference; TLC: total leucocyte count; CRP: C-reactive protein The p-value was calculated using Fisher’s exact test.

Variables	Fluid-responsive (n = 26)	Fluid-refractory (n = 27)	P-value
Age, months, median (IQR)	12 (9, 26)	18 (9, 26)	0.29
Male, n (%), n = 31	11(42.3)	20 (74)	0.03
Edema, n (%), n = 16	5 (19.2)	11(40.7)	0.13
Lethargic, n (%), n = 31	12 (46.1)	19 (70.3)	<0.0001
Oliguria, n (%), n = 28	9 (34.6)	19 (70.3)	0.01
Heart rate, mean (SD)	161.8 (9.15)	155.2 (16.14)	0.07
Respiratory rate, mean (SD)	53.8 (10.21)	57 (12.88)	0.32
MUAC, mean (SD)	9.86 (1.19)	9.83 (1.54)	0.93
Weight, mean (SD)	6.2 (2.52)	6.16 (2.64)	0.95
Hemoglobin, mean (SD)	9.38 (2.13)	9.2 (1.97)	0.78
TLC, median (IQR)	12,400 (8,530, 15,200)	15,200 (8,834, 20,500)	0.07
Urea, median (IQR)	24 (19.6, 30.5)	34 (22.2, 94.70)	0.01
Creatinine, median (IQR)	0.40 (0.40, 0.55)	0.60 (0.46, 1.1)	<0.0001
CRP, median (IQR)	3.1(2.3, 6.3)	23.3 (12.57, 37)	<0.0001
pH, mean (SD)	7.26 (0.05)	7.13 (0.25)	0.02
Lactate, median (IQR)	3.2 (1.9,4.4)	4.6 (3.2,6)	0.007
Diarrhea, n (%), n = 40	22 (84.6)	18 (66.6)	0.20
Pneumonia, n (%), n = 12	3 (11.5)	9 (3.3)	0.10
Sepsis, n (%), n = 30	5 (19.2)	25 (92.5)	<0.0001

**Table 3:** Resolution of features of shock at eight and 24 hours. *: Fisher’s exact test was used to compare the groups; @: fluid-refractory children who received a blood transfusion as well as inotropes; #: fluid-refractory children who did not receive a blood transfusion (n = 7) and fluid non-responsive children (n = 3);  $: 13 children expired (four at 24 hours, three at 48 hours, six within seven days) and all children had oliguria and shock; &: oliguria at eight hours

Variables	Total	Resolution of features of shock (hours)		P-value^*^
		At 8 (n=53)	At 24 (n=49)	
All, n (%)	53	10 (18.9)	35 (71.4)	<0.0001
Fluid-responsive, n (%)	26	10/26 (38.4)	24/26 (92.3)	<0.0001
Fluid-refractory^@^, n (%)	17	17/17 (100)	6/13 (46.1)	0.03
Inotrope(s)^#^, n (%)	10	10/10 (100)	5/10 (50)	0.03
Mortality^$^, n (%)	13	0	4/13 (30.7)	0.09
Oliguria^&^, n (%)	28	28/53 (52.8)	12/49 (24.4)	0.004

**Table 4:** Univariate analysis of risk factors associated with mortality. MUAC: mid-upper arm circumference; TLC: total leucocyte count; CRP: C-reactive protein; OR: odds ratio; CI: confidence interval @: variables expressed as frequency or mean (SD). P-values were calculated using Fisher’s exact test and variables are expressed as median (IQR). P-values were calculated using the Mann-Whitney U test.

Variables	Expired, n=13 (%)	Survived, n = 40 (%)	P-value^@^
Age, months, median (IQR)	16.5 (4.5, 25.5)	12.5 (9.25, 24)	0.95
Male, n = 13	7 (53.8)	24 (60)	0.75
Weight, median (IQR)	5.55 (2.25, 8.43)	6 (2.8,16)	0.39
MUAC, mean (SD)	9.39 (2.03)	9.98 (1.09)	0.20
Edema, n = 15	7 (53.8)	8 (20)	0.03
Lethargic, n = 39	12 (92.3)	27 (67.5)	0.01
Oliguria, n = 28	11 (84.6)	17 (42.5)	0.01
Blood transfusion, n = 17	10 (76.9)	7 (17.5)	0.002
Inotropes scores, median (IQR)	50 (42.5, 50)	10 (10, 40)	<0.0001
Mechanical ventilation, n = 10	9 (69.2)	1 (2.5)	<0.0001
Hemoglobin, g/dL, mean (SD)	9.4 (1.9)	9.2 (2.09)	0.96
TLC median (IQR)	15,300 (9,605.5, 21,200)	12600 (8,475, 18,000)	0.33
Urea, mg/dL, median (IQR)	48.1 (23.7,114.5)	26 (19.6,33.5)	0.03
Creatinine, median (IQR)	0.55 (0.45,1.82)	0.50 (0.40,0.60)	0.09
CRP, median (IQR)	28.1 (14.7, 37.8)	6 (2.8, 16)	0.001
pH, mean (SD)	7.03 (0.33)	7.24 (0.10)	0.001
Lactate, median (IQR)	4.6 (3, 6.35)	4.1 (2.13, 4.6)	0.11

## Discussion

A few studies have evaluated the efficacy of the WHO treatment protocol of shock in children with SAM. The reported literature shows heterogeneity in the type of fluid used, criteria of shock, the therapeutic endpoint of shock management, age group, and treatment [2,4,5]. However, this study used all four WHO criteria for the diagnosis of shock in children with SAM and assessed features of resolution of shock (absence of all; fast and weak pulse, CRT >3 seconds, urine output <0.5 mL/kg/hour, impaired consciousness) and decreasing trend of lactate and improving trend of pH. The present study demonstrated better results in fluid-responsive children compared to fluid-refractory children. This might be because fluid-refractory children were significantly lethargic, oliguric, uremic, septic, and acidotic compared to fluid-responsive children. Akech et al. [2] reported a randomized controlled trial that prematurely stopped. They included SAM children over six months old who presented with one or more of the following: CRT >2 seconds, lower limb temperature gradient, weak pulse volume, deep acidotic breathing, creatinine >80 µmol/L or decreased conscious state, and received ringer lactate (40 mL/kg over two hours) or half-strength Darrow’s solution (30 mL/kg over two hours) and assessed resolution of shock (absence of all; severe tachycardia PR >160/minute, CRT >2 seconds, or oliguria urine output <1 mL/kg/hour) and found poor outcomes in both treatment arms, with persistence of oliguria at eight and 24 hours. However, slightly better outcomes were noted with the isotonic Ringer’s lactate compared with the hypotonic half-strength Darrow’s solution. The important factors that differed from the present study were the criteria of shock, i.e., the presence of one or more of all, and only 41/61 (75%) children fulfilled the WHO criteria for the diagnosis of shock. The study did not use inotropes, mechanical ventilation, and hydrocortisone. We also observed less than 50% resolution of shock at 24 hours and 12 (52.1%) cases of persistence of oliguria and acidosis in fluid-refractory shock despite the use of inotropes, mechanical ventilation, and hydrocortisone at 24 hours. A randomized controlled trial by Oza et al. [5] from India compared the WHO guidelines (0.9% NaCl bolus at 30 mL/kg over two hours) and the Indian Academy of Pediatrics (IAP) guidelines (0.9% NaCl bolus at 30 mL/kg bolus over one hour) as initial bolus, followed by inotropes in case of fluid-refractory state. The study concluded significant improvement in shock at one hour (p = 0.02) in IAP guidelines, whereas 40% and 60% improvement in shock at 12 hours in WHO vs. IAP guidelines. However, there was no significant difference at 12 hours (p = 0.10). A study by Obonyo et al. [4] compared restricted (30 mL/kg over two hours) vs. liberal fluid (10/kg/hour over five hours without bolus) resuscitation and assessed clinical, hemodynamic, and echocardiographic data. They concluded cardiovascular collapse, i.e., the persistence of shock and hypovolemia. The above-mentioned studies and the present study suggested that restrictive and slow fluid resuscitation endorsed by the WHO is not able to expand the desired vascular volume to achieve improvement in shock after fluid bolus and suboptimal response of inotropes.

The reported case fatality in children with SAM with septic shock ranged from 40% to 69% [8,9]. In-hospital mortality in the present study was 13/53 (24.5%), and we observed mortality among fluid-refractory shock cases and found 4/23 (17.3%) deaths at 24 hours. All expired children during hospitalization had oliguria, acidosis, and shock. Akech et al. [2] reported 31/61 (51%) in-hospital mortality and 12/31 (39%) deaths at 24 hours. Further sub-group analysis revealed even higher mortality, 84% (26/31) in children who fulfilled the WHO criteria for diagnosis of shock. Obonyo et al. [4] reported 35% of deaths at 48 hours and higher deaths at 28 days (81.8%) and concluded that the persistence of shock was a cause of early death within 48 hours, with comorbidities contributing to mortality later. We observed similar results in fluid-refractory children in whom features of shock and oliguria were persisting. Moreover, these children presented with acute diarrhea and culture-positive sepsis. On univariate analysis, it was found that edema, oliguria, lethargy, blood transfusion, urea, C-reactive protein, mechanical ventilation, inotrope scores, and pH at admission were significantly associated with mortality. However, during multivariate analysis, it was found that children requiring mechanical ventilation had an 85 times increased chance of mortality. Inotropes score <20, i.e., the use of dopamine, led to favorable outcomes, whereas the requirement of epinephrine or dobutamine led to adverse outcomes. Interestingly, we found favorable outcomes in children who received a blood transfusion, which might be because almost all children who received blood also received inotropes. A retrospective analysis of 93 septic children with SAM and shock who received blood transfusions and inotropes [8] revealed that dopamine had a favorable outcome, and whenever additional vasopressor was required, it increased the probability of death. Previously published literature demonstrated that children who required blood transfusion for the management of crystalloid-resistant systolic hypotension were at risk of death [10-12], and it was thought that dysfunction of alveolar epithelial sodium and chloride transport in children with SAM impedes fluid clearance from the alveolar exudates [13,14] or transfusion-related acute lung injury [15]. The present study did not find blood transfusion as a risk factor because almost all children who received blood also received inotropes.

Our small sample size (fluid-refractory shock, n = 27), did not permit further subgroup analysis to compare blood transfusion and inotropes. Thus, further studies are required to assess the efficacy of blood transfusion vs. inotropes or individual inotropes in fluid-refractory shock. The strength of the present study was uniform inclusion criteria as well as features of resolution of shock. We preferred to calculate sample size based on mortality at 24 hours of hospitalization because mortality is a hard outcome and we deviated 29.1% from protocol. We had a few limitations in our study. We could not perform echocardiography, or cardiac biomarkers to estimate fluid overload, hypovolemia, myocardial dysfunction, or cardiac injury. Subgroup analysis had a limited sample which prevented us from achieving more statistical robustness. To assess the effect of individual inotrope(s) or blood transfusion, further randomized controlled trials are needed.

## Conclusions

The WHO protocol for the management of shock in children with SAM is effective in fluid-responsive shock, whereas fluid-refractory children were significantly lethargic, oliguric, uremic, septic, and acidotic compared to fluid-responsive children. There was less than 50% recovery from shock in fluid-refractory children, and those who required ventilation were at 85 times the risk of mortality. Inotrope score <20 and blood transfusion has a favorable outcome. However, further randomized controlled trials with adequate power are needed to assess the effect of individual inotrope or blood transfusions.
